# Mechanisms That Prevent Vascular Leakage During Leukocyte Extravasation

**DOI:** 10.1111/apha.70126

**Published:** 2025-11-02

**Authors:** Siem J. de Haan, Jaap D. van Buul

**Affiliations:** ^1^ Department of Medical Biochemistry, Amsterdam UMC University of Amsterdam Amsterdam the Netherlands; ^2^ Van Leeuwenhoek Centre for Advanced Microscopy, Section of Molecular Cytology Swammerdam Institute for Life Sciences at University of Amsterdam Amsterdam the Netherlands

**Keywords:** adhesion, diapedesis, endothelium, inflammation, migration, vasculature

## Abstract

**Background:**

Inflammation is the response of the immune system against harmful stimuli in tissues. Leukocyte extravasation or TransEndothelial Migration (TEM) is a crucial step during inflammation, in which leukocytes migrate over the endothelial barrier toward the damaged tissue.

**Objective:**

Historically, it was believed that leukocyte TEM directly causes excessive vascular leakage, resulting in tissue edema. However, it is now clear that leukocyte TEM and vascular leakage are uncoupled events with different spatiotemporal regulation. Moreover, several mechanisms have been identified that prevent vascular leakage during leukocyte TEM.

**Conclusion:**

Here we summarize the different mechanisms that are responsible for limiting the leakage during the transmigration event and explore their clinical relevance in developing targeted therapeutics for controlling vascular leakage in inflammatory diseases.

**Summary**

- Leukocyte Transendothelial Migration (TEM) is essential for inflammation but does not directly cause vascular leakage.
- Mechanisms like endothelial dome formation, ventral lamellipodia, and F‐actin rings prevent leakage by stabilizing endothelial adhesions.
- Understanding these processes offers insights for targeted therapies to control vascular leakage in inflammatory diseases.

## 1 Introduction

The vascular system is one of the vital systems in the human body that provides organs with nutrients and oxygen as a primary function, but also fulfills some important secondary tasks [1]. All these functions are essentially carried out by the blood, which is transported by the vascular system and composed of two major components. The first component is the plasma, which accounts for 55% of the total blood volume and contains essential compounds like proteins, electrolytes, coagulants and immunoglobulins [2]. The other 45% of the blood volume is known as the cellular component, and contains erythrocytes, leukocytes and platelets [3]. Although leukocytes represent the cell population with the lowest abundance in the circulation, they play a crucial role in the immune system, which is facilitated by the vascular system and the connected lymphatic system [1].

Leukocytes can be subdivided into five different subtypes that uniquely contribute to the immune system [4, 5]. Neutrophils typically respond to bacterial infections by rapidly killing and phagocytizing damaged tissue. This innate immune response is assisted by another leukocyte type, as monocytes differentiate into macrophages and phagocytose the damaged tissue as well. In addition, monocytes differentiate into dendritic cells to present pathogen‐specific antigens to lymphocytes in the lymph nodes. These lymphocytes constitute the third type of leukocytes in the blood, and are responsible for the adaptive immune response. This slower immune response is mediated by B‐lymphocytes producing pathogen‐specific antibodies and T‐lymphocytes coordinating the targeted killing of damaged cells. Natural killer (NK) cells also belong to the lymphocytes, but mainly play a role in the innate immune response against infections and tumors [6]. The leukocyte population is completed by eosinophils and basophils, both of which are involved in allergic reactions. In addition, eosinophils contribute to the immune response against parasites [7].

Inflammation is the overarching process in which all these leukocyte subtypes are generally involved. By definition, inflammation is the response of the immune system against harmful stimuli in tissues. Harmful stimuli can be categorized as either infectious stimuli, such as bacteria and viruses, or non‐infectious stimuli, such as trauma and toxic chemicals [8]. Upon exposure to harmful stimuli, the inflammatory cascade is rapidly initiated within the tissue, including local vasodilatation, plasma leakage and transendothelial migration (TEM) of leukocytes across the endothelium toward the damaged tissue [9]. This response has been shaped by evolution to quickly restore tissue structure and functionality [10]. However, as the human life expectancy has been rapidly increasing over the last centuries, this inflammatory response has paradoxically become one of the major challenges in human medicine. In fact, it is a chronic level of inflammation that forms the basis of many prevalent diseases today. One problematic hallmark of inflammatory diseases is excessive plasma leakage, which occurs in acute diseases such as sepsis [11] and coronavirus disease 2019 (COVID‐19) [12], but also in chronic diseases like atherosclerosis [13] and inflammatory bowel disease (IBD) [14].

TEM of leukocytes has long been considered the reason for excessive plasma leakage in various inflammatory diseases [15]. This hypothesis was based on the assumption that leukocytes damage the endothelial wall during TEM, creating endothelial gaps that lead to leaky blood vessels. However, later investigations showed that vascular leakage does not necessarily happen during leukocyte TEM. In fact, it was demonstrated that vascular leakage is actively prevented by several mechanisms during leukocyte TEM [16]. Because these mechanisms were discovered in different studies, the aim of this review is to provide an integrated overview of the mechanisms that prevent vascular leakage during leukocyte TEM.

To clarify these mechanisms, we first offer a brief introduction to endothelial cell biology. Next, we present the multi‐step model of leukocyte TEM and its presumed association with vascular leakage. Building on this, we discuss the possible mechanisms that prevent vascular leakage during leukocyte TEM. The review concludes with a section discussing the clinical implications of these mechanisms for advancing the prevention of vascular leakage in various diseases.

## 2 The Biology of Endothelial Cells

The human vascular system can be divided into five major blood vessel types [17]. The large arteries, along with the smaller arterioles they branch into, are responsible for the transport of blood from the heart to all tissues. Within tissues, the arterioles branch into the capillaries that orchestrate the exchange of metabolites and fluids between the intravascular space and the surrounding tissue, which continues as the capillaries drain into the venules. When these venules finally merge into the larger veins, the exchange between the blood and surrounding tissue is no longer possible and the blood is returned to the heart. Except for the capillaries, these vessels are composed of three distinct layers. The outermost layer is known as the tunica adventitia, and supplies the blood vessel with structural integrity. The middle layer, referred to as the tunica media, regulates the diameter of the blood vessel by its elastic and muscular tissue. Finally, the inner layer is defined as the tunica intima, which provides the luminal side of the blood vessel with a continuous surface of endothelium that directly faces the blood.

The endothelium is made up of thin and slightly elongated endothelial cells that are interconnected by lateral cell–cell junctions [18]. Endothelial cells typically display an apical side exposed to the lumen of the vessel, and a basolateral side oriented in the opposite direction toward the tissue. On the apical side, endothelial cells interact with intravascular components using their membrane receptors and a mesh‐like framework called the glycocalyx [19]. On the basolateral side, endothelial cells produce a collagen‐rich basement membrane, which mainly provides mechanical support to the endothelial layer [20]. Embedded in this basement membrane, pericytes contribute to the integrity of the endothelial barrier by engaging in paracrine and juxtacrine communication with endothelial cells [21]. Importantly, the endothelial layer and basement membrane constitute the only barrier between the intravascular space and the surrounding tissue in capillaries [17]. In addition, the endothelial layer and basement membrane also form the predominant barrier in venules, which typically have a very thin tunica media and tunica adventitia. Therefore, endothelial cells have a critical role in preventing unnecessary vascular leakage from these blood vessel types [22].

The actin cytoskeleton of endothelial cells is of vital importance for their function as a vascular barrier [23]. This three‐dimensional actin network has a highly dynamic character, allowing endothelial cells to form cell–cell adhesions with their neighbors [24] and cell‐matrix adhesions with the extracellular matrix (ECM) [25], the latter primarily involving the basement membrane. At the molecular level, the actin cytoskeleton is composed of filamentous actin (F‐actin), a polymer that can be formed by polymerization of type ß and type γ globular actin (G‐actin) in a double helical fashion [26]. Polymerization of F‐actin occurs in two steps [27], starting with the nucleation of three G‐actin monomers that bind together in a specific conformation. This conformation initiates the second step of polymerization, which is the elongation of the filament using ATP‐bound G‐actin monomers. This elongation step is coordinated by a complex machinery that involves the recruitment of the actin‐related protein 2/3 complex (Arp2/3) by local proteins like cortactin [28].

**FIGURE 1:**
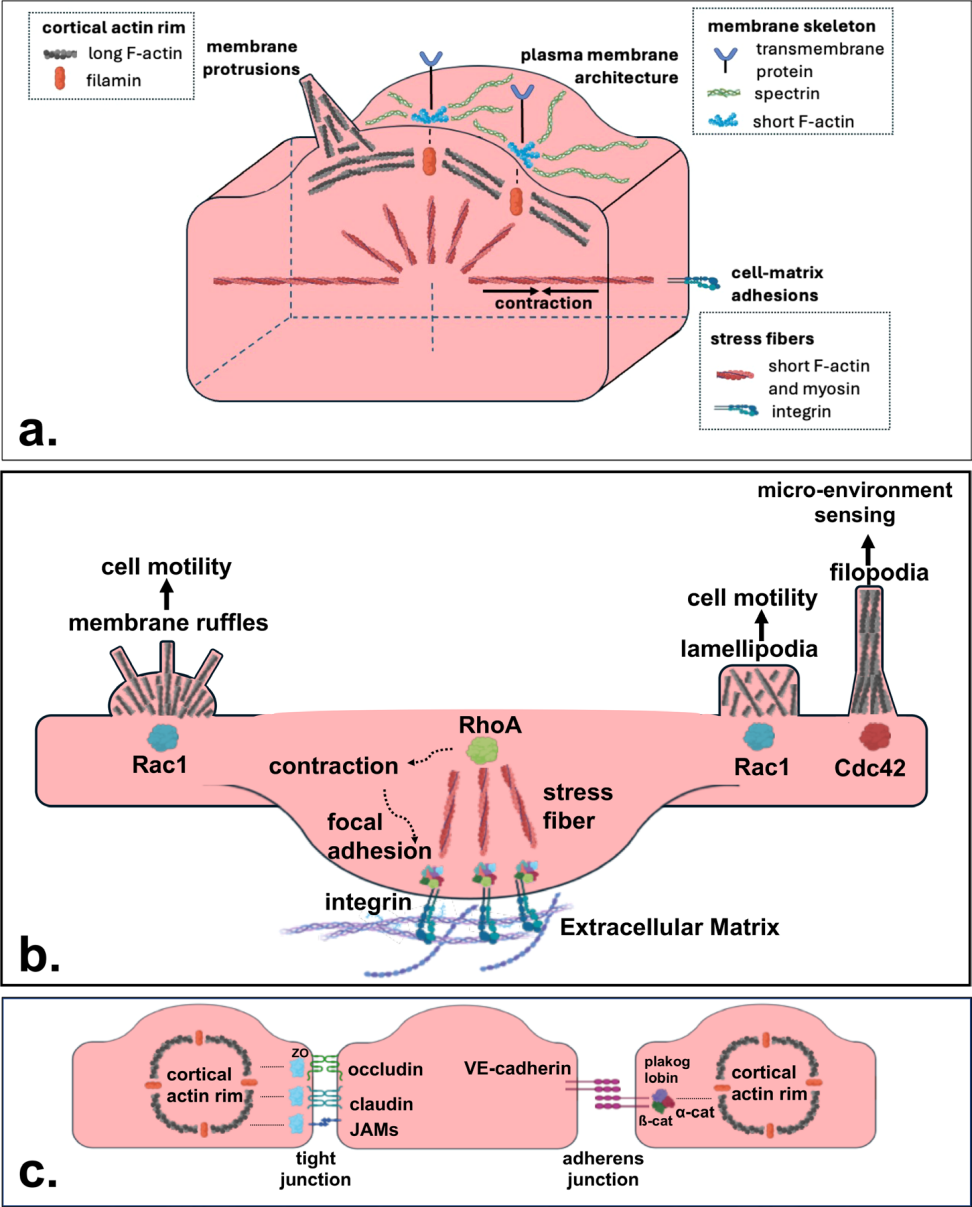
The cytoskeleton of the endothelial cell and its interactions. (a) Schematic overview of the cytoskeleton of endothelial cells, composed of stress fibers, the cortical Actin rim and the membrane skeleton. (b) Schematic overview of endothelial cell‐matrix interactions, regulated by three Rho GTPases. RhoA activates contraction (arrows) of stress fibers, which assembles focal adhesions at the intracellular side of integrin receptors. In turn, these focal adhesions induce stress fiber formation, which links the ECM to the endothelial Actin cytoskeleton via integrin receptors. Rac1 is involved in the formation of lamellipodia and membrane ruffles. Cdc42 is involved in the formation of filopodia. (c) Schematic overview of endothelial cell–cell interactions, regulated by tight junctions and adherens junctions. Tight junctions are constructed by the membrane proteins occludin, claudin and junctional adhesion molecules (JAMs), which are anchored to the Actin cytoskeleton by zonula occludens proteins (ZO). Adherens junctions are constructed by the glycoprotein vascular endothelial cadherin (VE‐cadherin), which binds plakoglobin and ß‐catenin on its intracellular tail and is connected to the Actin cytoskeleton via α‐catenin. Created with Biorender.com.

Three important actin cytoskeletal structures have been defined in cell biology [29] (Figure 1a). The first structure is the membrane skeleton, a solid network of spectrin located directly under the plasma membrane [30]. This structure regulates the architecture of the plasma membrane via short F‐actin filaments that crosslink the network of spectrin to transmembrane proteins. Directly underneath the membrane skeleton, the cortical actin rim can be found as the second important actin structure [31]. This rim contains a dense network of long F‐actin filaments, which are crosslinked to the membrane skeleton by proteins such as filamins and constitute the reservoir for F‐actin‐driven membrane protrusions [32]. The third actin structure is constituted by the stress fibers, organized in bundles of short F‐actin and myosin that radiate throughout the cell. Stress fibers mediate cell‐matrix adhesions and also allow the cell to perform tension‐mediated contraction [33]. Together, these three structures of the actin cytoskeleton regulate the endothelial barrier by supporting endothelial cell–cell and cell‐matrix adhesions.

Cell–cell adhesions are formed by lateral junctions that connect endothelial cells. In general, these junctions are composed of tight junctions and adherens junctions, both of which are dynamically regulated by the actin cytoskeleton [34] (Figure 1b). Tight junctions are constructed by several membrane proteins, including claudin, occludin and junctional adhesion molecules (JAMs) [35]. These membrane proteins firmly bind neighboring cells together, mainly by homophilic adhesions of their extracellular heads. On the intracellular side, their cytosolic tail attaches to the actin cytoskeleton via multiple variants of the zonula occludens (ZO) protein [36]. Adherens junctions, on the other hand, are built of only one component known as the glycoprotein vascular endothelial (VE) cadherin. The extracellular heads of VE‐cadherin are also involved in homophilic interactions to establish a stable junction, albeit one that is weaker than the tight junction. It is noteworthy that these tight junctions are much better developed and functional in the endothelium of the brain, being a critical component of the blood–brain barrier. On the intracellular side, the tails of VE‐cadherin dimerize to form VE‐cadherin clusters [37]. Moreover, these tails also bind ß‐catenin and plakoglobin to anchor on to the actin cytoskeleton via α‐catenin [38].

Cell‐matrix adhesions are formed by interactions between the endothelial cell membrane and the extracellular matrix (ECM). These adhesions are controlled by small GTPases of the Rho family, of which RhoA, Rac1 and Cdc42 are the most studied ones [39] (Figure 1c). RhoA promotes contraction of endothelial stress fibers via its effector Rho‐kinase (ROCK) [40]. To be specific, ROCK phosphorylates the myosin light chain (MLC) of stress fibers directly [41], and also indirectly by inhibiting myosin phosphatase [42]. This results in stress fiber contraction, which drives the assembly of focal adhesions on the intracellular side of integrins on the endothelial membrane [43]. Focal adhesions are protein complexes that form stress fibers and anchor the cytoskeleton to the ECM via integrins. Although focal adhesions stabilize the endothelial barrier in principle [44], pro‐inflammatory signals like tumor necrosis factor‐alpha (TNFα) can amplify stress fiber contraction to a level that disrupts the endothelial junctions, resulting in vascular leakage [45]. In contrast to RhoA, Rac1 and Cdc42 mainly seem to stabilize the endothelial barrier [46]. Rac1 induces lamellipodia and membrane ruffles which are involved in cell motility [47], whereas Cdc42 initiates thin filopodia that function as sensors of the microenvironment [48].

## 3 Transendothelial Migration of Leukocytes

Leukocyte interactions with the endothelial cell monolayer do not occur under healthy conditions. In fact, such interactions have only been shown for a subset of monocytes and T‐cells that patrol blood vessels by crawling along the endothelial wall [49]. However, when inflammation occurs in a tissue, the clearance of damaged cells and pathogens by leukocytes is of crucial importance for the structural and functional recovery of the tissue. In this case, leukocytes and endothelial cells intensify their interactions to stimulate leukocyte migration from the blood vessel toward the damaged tissue. This interactive process has been characterized as a multistep cascade, originally outlined in two landmark papers from the 90's [50, 51]. The final step of this cascade model is the actual migration of the leukocyte across the endothelial cell monolayer. This step, known as diapedesis, can proceed via two distinct routes [52]. The predominant route is the paracellular pathway, where leukocytes migrate through the lateral junctions of endothelial cells. Alternatively, leukocytes can also migrate directly through the cell body of the endothelial cell, which is known as the transcellular pathway.

Because the paracellular route is considered the most important one, this review will be focused on the dynamics of paracellular TEM. However, this does not exclude the transcellular pathway from being important or relevant. In fact, subsets of T‐cells prefer the transcellular pathway over the paracellular route [53]. Moreover, transplanted hematopoietic stem and progenitor cells can switch between both pathways during their homing to the bone marrow, depending on the availability of each route [54]. For more details, we refer the reader to excellent reviews on transcellular TEM and its potential relevance and regulation [55, 56]. Still, the majority of leukocytes seem to prefer the paracellular route. To get a better understanding of the interactions between leukocytes and endothelial cells during paracellular TEM, it is important to first describe the equipment that both cell types need to achieve a successful event of leukocyte TEM.

## 4 TEM Equipment of the Endothelial Cell

### 4.1 Selectins

Selectins are transmembrane glycoproteins from the lectin family [57]. Three variants of selectin have been denoted in the literature, including E‐selectin, P‐selectin, and L‐selectin. Following exposure to pro‐inflammatory stimuli, endothelial cells start synthesizing E‐selectin [58] and mobilizing P‐selectin from certain Weibel‐Palade bodies to express both of these selectins on their apical membrane [59]. Selectins are expressed on microvillus‐like projections and interact with P‐selectin glycoprotein ligand 1 (PSGL1) and L‐selectin on the leukocyte [60]. These interactions typically control the first part of the TEM cascade, known as the rolling phase.

### 4.2 Integrin Ligands

Intercellular adhesion molecule 1 (ICAM‐1), intercellular adhesion molecule 2 (ICAM‐2), and vascular cell adhesion molecule 1 (VCAM‐1) are known as integrin ligands. They belong to the immunoglobulin superfamily, as they contain homologous domains of immunoglobulins [61]. Endothelial expression of ICAM‐1 and VCAM‐1 is strongly upregulated by pro‐inflammatory stimuli [62]. Upon binding to leukocyte integrins, ICAM‐1 and VCAM‐1 cluster on the apical membrane and trigger intracellular signaling, which couples the intracellular domain of these integrin ligands to actin regulators such as α‐actinin, cortactin and filamin. Together with the local activation of small GTPases, this results in the formation of membrane protrusions that extend the endothelial membrane surface [63]. In contrast to ICAM‐1 and VCAM‐1, ICAM‐2 is already expressed on the apical membrane and lateral junctions of endothelial cells without exposure to pro‐inflammatory agents [64]. ICAM‐2 also facilitates efficient leukocyte TEM, possibly by mediating the migration behavior of leukocytes against the direction of the flow [65]. Moreover, research on the endothelial blood–brain barrier revealed that ICAM‐2 partners up with ICAM‐1 to mediate the crawling of leukocytes, which follows leukocyte arrest during TEM [66].

### 4.3 Chemokines

Chemokines are chemotactic cytokines that constitute a family of roughly 50 small proteins [67]. They are produced by numerous cell types within inflamed tissues, including endothelial cells, and reside on the apical surface of endothelial cells bound to glycosaminoglycans in the glycocalyx [68]. During inflammation, chemokines mark vessels that surround the damaged tissue to recruit leukocytes [69]. Besides this traditional function, chemokines from the CXCL and CCL families also interact with G‐protein coupled receptors (GPCRs) on the leukocyte to regulate the activity of their integrins [70]. This activation occurs during the rolling of leukocytes and marks the start of leukocyte arrest.

### 4.4 VE‐Cadherin

VE‐cadherin is a glycoprotein exclusively present at the lateral junctions of endothelial cells, and regulates leukocyte TEM in a negative way [71]. Expression of VE‐cadherin is dependent on the phosphorylation status of the whole VE‐cadherin–catenin complex [72]. Under normal conditions, the transmembrane protein vascular endothelial‐protein tyrosine phosphatase (VE‐PTP) associates with VE‐cadherin and retains the entire VE‐cadherin–catenin complex in a dephosphorylated state. In addition, the catenin p120 binds the intracellular tail of VE‐cadherin, preventing clathrin‐mediated endocytosis of VE‐cadherin [73]. Pro‐inflammatory agents and leukocyte adhesion result in the dissociation of VE‐PTP from VE‐cadherin [72], leading to phosphorylation of the VE‐cadherin–catenin complex by tyrosine kinases downstream of ICAM‐1 and VCAM‐1 clustering [74, 75]. This phosphorylation decreases the binding affinity of p120 for VE‐cadherin. As a result, the clathrin‐dependent endocytosis of VE‐cadherin is no longer inhibited, and VE‐cadherin is internalized to the cytosol. This process is critical for the initiation of diapedesis, the final step of leukocyte TEM.

### 4.5 Other Adhesion Molecules in the Cell–Cell Junctions

Several other adhesion molecules and receptors also play a role during diapedesis, including platelet endothelial cell adhesion molecule 1 (PECAM‐1) [76], junctional adhesion molecules (JAMs) [77], endothelial cell‐selective adhesion molecule (ESAM) [78], CD99 [79] and CD99L2 [80]. Under normal circumstances, these adhesion molecules and receptors are already expressed at the lateral junction of endothelial cells, where they mainly engage in homophilic interactions with their equivalents on the membrane of neighboring endothelial cells. In diapedesis, however, these bindings are redirected to form connections between endothelial cells and leukocytes, supporting the passage of the leukocyte [81]. Interestingly, PECAM‐1, JAM‐A and CD99 are continuously recycled by a vesicle‐like lateral border recycling complex (LBRC) during diapedesis, underlining the importance of these mediators in leukocyte TEM [82].

## 5 TEM Equipment of the Leukocyte

### 5.1 Selectins

Two selectins are also upregulated on leukocytes in inflamed vessels, known as P‐selectin glycoprotein ligand 1 (PSGL1) and L‐selectin. PSGL1 is a homodimeric membrane protein with an extracellular domain that resembles a mucin [83], and is the most important ligand for P‐selectin on endothelial cells during inflammation [84]. L‐selectin is a membrane glycoprotein that binds E‐selectin on inflamed endothelial cells and also binds P‐selectin, but with a lower affinity than PSGL1 [85]. Both PSGL1 and L‐selectin are mediators of the rolling phase during leukocyte TEM.

### 5.2 Integrins

Integrins are membrane proteins that link the actin cytoskeleton to the ECM [86]. Because integrins are heterodimers composed of variable a‐ and ß‐subunits, 24 different integrins have been described in vertebrates [87]. Three of these integrins are involved in leukocyte TEM [88], including lymphocyte‐associated antigen 1 (LFA1 or αLß2) which is present on all leukocytes; macrophage antigen 1 (MAC1 or αMß2) which is present on all leukocytes except lymphocytes; and very late antigen 4 (VLA4 or α4ß1), which is present on lymphocytes, monocytes, and eosinophils [89]. Under normal conditions, integrins reside in a bent extracellular conformation on the leukocyte membrane, resulting in a low affinity for their ligands. However, chemokines and transient contacts with endothelial selectins modify this extracellular domain to an intermediate‐affinity state by extending its conformation, and subsequently to a high‐affinity state by opening its ligand‐binding head [90]. Mechanistically, this is achieved by activating intracellular proteins such as talin and kindlin, which increase the distance between the intracellular legs of the integrin α‐ and ß‐subunit [91, 92]. In addition, these proteins also mediate the clustering of integrins by the actin cytoskeleton, resulting in firm binding to endothelial integrin ligands that mark the leukocyte arrest phase [93].

### 5.3 Other Adhesion Molecules and Receptors

Some adhesion molecules and receptors on endothelial cells can also be found on leukocytes, including PECAM‐1, CD99 and CD99L2 [81]. This dual expression facilitates homophilic binding between endothelial cells and leukocytes, which is essential during the diapedesis phase of leukocyte TEM.

## 6 The Cascade of Leukocyte TEM

**FIGURE 2:**
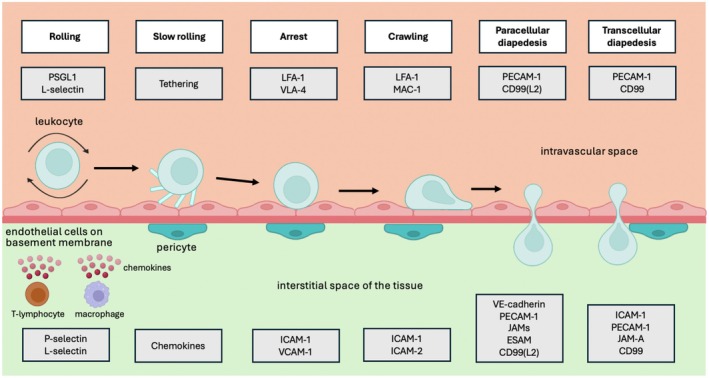
Transendothelial migration of leukocytes. Schematic overview of leukocyte TEM. Following damage in a tissue, local cells produce pro‐inflammatory stimuli that lead to upregulation of P‐selectin and L‐selectin on the endothelial membrane. These selectins capture leukocytes by making transient contacts with PSGL1 and L‐selectin on the leukocyte membrane, resulting in rolling of the leukocyte along the endothelial wall. Subsequently, the leukocyte membrane forms tethers that slow down the rolling, while chemokines produced by endothelial cells activate integrins on leukocytes at the same time. As a result, LFA‐1/ICAM‐1 or VLA‐4/VCAM‐1 interactions lead to the arrest of leukocytes on the endothelial wall. Following arrest, leukocytes start crawling along the vessel wall using LFA‐1/ICAM‐1 interactions or MAC‐1/ICAM‐2 interactions. From this point, the leukocyte can enter the irreversible step of diapedesis via two routes. In the paracellular pathway, inflammatory stimuli and leukocyte adhesion lead to dissociation of VE‐cadherin from the endothelial cell–cell junction. This enables the leukocyte to start migrating through the endothelial cell–cell junction. During this process, several adhesion molecules and receptors become involved on the endothelial cell, including PECAM‐1, JAMs, ESAM, CD99 and CD99L2. Three of these bind their equivalent on the leukocyte membrane, whereas the ligand of others remains enigmatic. Besides the paracellular pathway, leukocytes can also complete TEM by transcellular diapedesis. Although this pathway is not of primary interest in this review, we have included the involved mediators that are described in another excellent review [96]. On the endothelial side, ICAM‐1, JAM‐A, PECAM‐1 and CD99 play a role in the transcellular pathway, whereas PECAM‐1 and CD99 are involved at the leukocyte. CD99‐L2, CD99 antigen‐like protein 2; ESAM, endothelial cell‐selective adhesion molecule; ICAM‐1, intercellular adhesion molecule 1; ICAM‐2, intercellular adhesion molecule 2; JAM‐A, junctional adhesion molecule‐A; JAMs, junctional adhesion molecules; LFA‐1, lymphocyte‐associated antigen 1; MAC‐1, macrophage antigen 1; PECAM‐1, platelet endothelial cell adhesion molecule 1; PSGL1, *P*‐selectin glycoprotein ligand 1; VCAM‐1, vascular cell adhesion molecule 1; VE‐cadherin, vascular endothelial cadherin; VLA‐4, very late antigen 4. Created with Biorender.com.

When a harmful stimulus causes damage in a tissue, resident cells like macrophages, mast cells, and T‐cells respond with the production of pro‐inflammatory agents [94, 95]. These agents trigger the expression of E‐selectin and P‐selectin on the endothelial membrane, which capture the leukocyte by making transient contacts with PSGL1 and L‐selectin, leading to the rolling of the leukocyte [84, 85] (Figure 2). As a result of these transient contacts, small parts of the leukocyte membrane detach from its cytoskeleton, forming tethers that slow down the rolling [97]. Simultaneously, chemokines bound to the endothelial membrane stimulate the activation and clustering of integrins on leukocytes via GPCRs [70]. This results in LFA‐1/ICAM‐1 interactions or VLA‐4/VCAM‐1 interactions between leukocytes and endothelial cells, depending on the leukocyte subtype [88]. As this process continues, integrins gradually develop their ligand binding sites from an intermediate‐affinity state to a high‐affinity state [92], which enables endothelial cells to arrest leukocytes on the endothelial wall.

Following this arrest, leukocytes start crawling along the vessel wall using MAC‐1/ICAM‐2 interactions [64] or LFA‐1/ICAM‐1 interactions [98], again depending on the leukocyte subtype. When a leukocyte arrives at the lateral junction of endothelial cells, the final and irreversible step of diapedesis can be initiated. At this point, the cell–cell junction is opened by dissociation of VE‐PTP from VE‐cadherin, which is triggered by the physical adhesion of the leukocyte and the downstream signaling of endothelial receptors that respond to pro‐inflammatory stimuli [72]. As soon as the leukocyte starts migrating into the junction, several adhesion molecules and receptors become involved in the diapedesis process. Importantly, some of these mediators play a role at a specific level of the diapedesis process [96]. For example, the early passage of leukocytes through the apical side of the junction is thought to be mainly regulated by ICAM‐2, whereafter passage through the middle section of the junction has been attributed to JAM‐A, and passage through the basement is believed to be mainly dependent on PECAM‐1, CD99 and CD99 antigen‐like protein 2 (CD99L2).

However, such a multi‐step model of diapedesis provides an oversimplification of the complex interplay between these adhesion molecules and receptors in reality. As diapedesis is still the most puzzling step in leukocyte TEM, reductionist models should be considered with caution. For instance, blocking one domain of the PECAM‐1 adhesion molecule results in leukocyte accumulation at the basement membrane, whereas blocking another domain of PECAM‐1 leads to the accumulation of leukocytes on the apical side, suggesting that PECAM‐1 is involved at multiple levels of diapedesis [99]. In addition, the adhesion molecule ESAM has not been attributed to a specific level of diapedesis, but its crucial role in opening endothelial cell junctions is well established [78]. The same is true for JAM‐C, which is known as an important determinant of the irreversible character of diapedesis [52]. Altogether, the specific mechanisms by which these adhesion molecules and receptors mediate diapedesis largely remain to be elucidated, although one might argue that one receptor may cover multiple actions, making it less specific than originally believed. For now, recognizing their collective role in the final step of leukocyte TEM is sufficient to grasp the upcoming sections of this review.

## 7 Leukocyte TEM and Vascular Leakage: Coupled or Uncoupled?

Inflammation involves the vasodilatation of local blood vessels, leakage of plasma and migration of leukocytes across the vascular endothelium toward the damaged tissue [9]. Vasodilatation and plasma leakage occur directly in response to a harmful stimulus, whereas TEM of leukocytes develops more gradually to exert a prolonged effect during inflammation [81]. The rapid induction of vasodilatation and plasma leakage is particularly important during infections, because the plasma contains complement proteins and antibodies that contribute to the clearance of micro‐organisms and viruses [100]. To permit leakage of these essential vascular components, local cells produce pro‐inflammatory agents to enhance the permeability of the vascular barrier, such as thrombin, bradykinin, histamine and vascular endothelial growth factor (VEGF) [101]. These agents bind their cognate receptors on the endothelial membrane and cause an intracellular rise of calcium. Consequently, myosin light chain kinase (MLCK) phosphorylates stress fiber myosin, resulting in the contraction of stress fibers that disrupts the endothelial junction and leads to plasma leakage.

Although the transient leakage of plasma is generally considered a beneficial mechanism to restore tissue functionality, an excessive level of plasma leakage can lead to severe edema with serious consequences for the functionality of the tissue [15]. Leukocytes have been recognized as the triggers for such an uncontrolled extent of plasma leakage. This was based on the observation that they disrupt the endothelial barrier under inflammatory circumstances. However, the hypothesis that leukocyte‐endothelial interactions and vascular leakage are coupled has been thoroughly debated over the last decades, as several studies also found evidence that both these processes are in fact uncoupled. In the next sections, we shed light on this debate by describing the traditional and modern views on the (un)coupling between leukocyte TEM and vascular leakage.

### 7.1 Traditional View

In the traditional view of vascular leakage during inflammation, researchers in the 1980s proposed that white blood cells, also called leukocytes, play a direct role in weakening the blood vessel wall. This idea came from experiments showing that when neutrophils, a major type of leukocyte, were depleted in animals, the amount of leakage from inflamed vessels was strongly reduced [102]. This suggested that neutrophils themselves were actively responsible for causing the vessel barrier to become leaky.

At the time, several possible mechanisms were put forward. One possibility was that neutrophils release proteolytic enzymes, which can break down proteins in the endothelial cell junctions, thereby compromising the tight seal between the cells [103]. Another proposed mechanism was that neutrophils could produce bioactive substances that stimulate the contraction of endothelial cells, which would lead to gaps forming between them [104]. A third suggested explanation was that the very act of neutrophils crossing the vascular wall directly disrupts the integrity of the endothelial barrier [105].

Support for the role of TEM came from studies showing that blocking β‐integrins on leukocytes significantly reduced vascular leakage in conditions of inflammation and ischemia–reperfusion injury [106]. Endothelial ICAM‐1, an important ligand for β‐integrins, was also shown to enhance vascular leakage through its downstream signaling pathways. Since integrins and ICAM‐1 were originally described as adhesion molecules that enable leukocytes to attach and migrate across the endothelium, these findings suggested that leukocyte transmigration itself contributes to leakage by creating transient physical gaps in the vessel wall.

In addition to these mechanical effects, it was also proposed that neutrophils could enhance permeability by producing reactive oxygen species (ROS) during or after TEM [107]. ROS can injure endothelial cells, damage their junctional proteins, and thereby increase local leakage. Together, these observations formed the early concept that leukocytes are not just passive responders to inflammation but active mediators of vascular barrier dysfunction.

### 7.2 Updated View

However, over the following decades, this *leukocyte‐centric model* began to be reassessed. Advances in imaging, genetic tools, and in vitro systems revealed that inflammatory vascular leakage can occur even in the absence of neutrophils or active TEM. The traditional view on the coupling between leukocyte TEM and vascular leakage was largely based on studies that considered blood vessels as a whole entity. Importantly, these studies were indeed able to establish an association between leukocyte TEM and vascular leakage, but lacked crucial information regarding the location and timing of both processes. This spatiotemporal knowledge was acquired in later studies, the first of which investigated the allergic response of tracheal vessels in rats [108]. Using electron microscopy, this study unraveled that plasma leakage mostly occurs in postcapillary venules, whereas leukocyte TEM predominantly takes place in the collecting venules without any signs of vascular leakage. This spatial discrepancy was also found in an aseptic cutaneous wound model, showing that vascular leakage takes place in the center of wounds whereas leukocyte TEM occurs at wound edges [109]. Moreover, this study also established a temporal discrepancy between leukocyte TEM and vascular leakage, as vascular leakage peaked substantially earlier than leukocyte TEM.

More evidence for the uncoupling of leukocyte TEM and vascular leakage was found in a study that investigated VE‐cadherin, the main regulator of the adherens junction between endothelial cells [110]. Interestingly, this study found that pro‐inflammatory agents initiate vascular leakage by phosphorylation of the tyrosine residue on position 685, whereas leukocyte TEM triggers the opening of the endothelial junction via dephosphorylation of the tyrosine residue on position 731. Altogether, this striking evidence formed the basis of the modern view that leukocyte TEM and vascular leakage occur via different molecular mechanisms. Compared to the traditional view, however, some compatibility may exist. For instance, the observation that leukocytes produce ROS during TEM to increase vascular permeability could still be valid [107], although these ROS then have to act upstream of the collecting venules to induce vascular leakage in the postcapillary venules. The ability of ROS to bridge such a spatial difference has already been demonstrated, as circulating leukocytes are able to induce vascular leakage by producing ROS without adherence to the endothelial wall [111].

## 8 Mechanisms That Prevent Vascular Leakage During Leukocyte TEM

Based on the previous section, it can be concluded that leukocyte TEM and vascular leakage are uncoupled events. However, this conclusion does not explain how vascular leakage is prevented during leukocyte TEM. Over the past decades, several mechanisms have been identified that limit vascular leakage during leukocyte TEM. These mechanisms will be discussed in the section below.

### 8.1 Endothelial Domes

Endothelial domes were the first mechanism suggested to prevent vascular leakage during leukocyte TEM. These domes were detected using in vivo electron microscopy, and described as thin apical endothelial membranes that encapsulated 72% of all adherent leukocytes on the endothelium [112]. Based on a similar morphology, these membranes were recognized as ICAM‐1‐ and VCAM‐1‐rich docking structures [63] or transmigratory cups [113]. Importantly, endothelial domes did not perform phagocytosis, as leukocytes trapped within an endothelial dome never entered the cytosol of endothelial cells, but migrated toward the tissue via the paracellular or transcellular pathway instead. Therefore, it was suggested that endothelial domes function as an effective sealing mechanism, preventing leakage of vascular components before the final step of diapedesis is initiated. Because the domes were assembled by thin protrusions of the endothelial membrane, it was hypothesized that endothelial domes are formed by cytoskeletal rearrangements in the cell.

**FIGURE 3:**
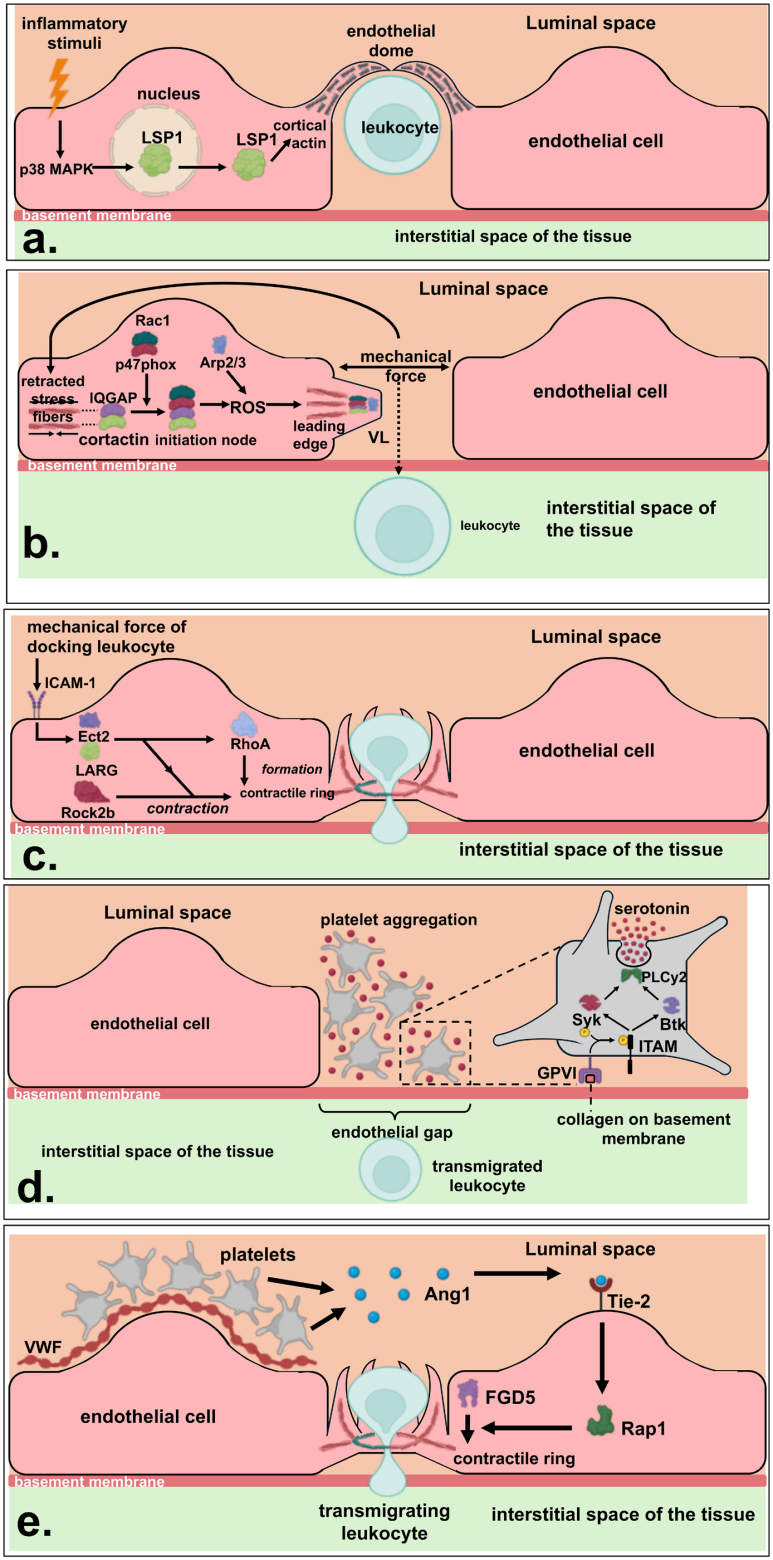
Mechanisms that prevent vascular leakage during leukocyte TEM. (a) Inflammatory stimuli activate the p38 MAPK pathway in endothelial cells, which induces translocation of LSP1 from the nucleus to the cytoskeleton. Following translocation, LSP1 regulates the formation of endothelial domes that encapsulate the leukocyte before diapedesis is initiated. (b) The mechanical force of leukocyte TEM evokes retraction of stress fibers in the endothelial actin cytoskeleton, resulting in activation of Rac1. Subsequently, Rac1 activates p47phox to produce ROS in the vicinity of the actin cytoskeleton, which is mediated by the linker proteins IQGAP and cortactin. The local production of ROS attracts Arp2/3 to the cytoskeleton, resulting in the formation of VL that bridge the junctional gap. (c) ICAM‐1 clustering during leukocyte TEM attracts the Rho GEFs LARG and Ect2 to the intracellular tail of ICAM‐1. Subsequently, these RhoGEFs activate RhoA to regulate the formation of an F‐actin‐rich contractile ring. Contraction of the F‐actin‐rich contractile ring is mediated by asymmetric myosin phosphorylation, which is mediated by ROCK2b and assisted by downstream signaling of Ect2 and LARG. (d) Platelets bind exposed sites of the collagenous basement membrane using their GPVI receptor, which results in phosphorylation of the ITAM domain of the Fc receptor y‐chain. Consequently, the tyrosine kinases Syk and Btk are activated and induce the degranulation of serotonin, resulting in platelet aggregation on the basement membrane. (e) Platelets dock to inflamed endothelial cells using a layer of VWF on the endothelium and produce Ang1 to stimulate the Tie‐2 receptor. This results in activation of Rap1, which attracts FGD5 to the endothelial junction. FGD5, in turn, contributes to the formation of an F‐actin‐rich contractile ring. Ang1, angiopoietin‐1; Arp2/3, Actin‐Related Protein 2/3 Complex; Btk, Bruton's tyrosine kinase; GPVI, Glycoprotein VI; IQGAP, IQ Motif Containing GTPase‐Activating Protein; ITAM, immunoreceptor tyrosine‐based activation motif; LARG, leukemia‐associated Rho guanine nucleotide exchange factor; LSP1, leukocyte‐specific protein 1; MAPK, mitogen‐activated protein kinase; ROCK2b, Rho‐associated protein kinase 2b; Syk, spleen tyrosine kinase; VWF, Von Willebrandfactor. Created with Biorender.com.

The mechanism by which endothelial domes are formed to prevent vascular leakage during leukocyte TEM has been elucidated in later studies. Endothelial domes were found to be dependent on the p38 mitogen‐activated protein kinase (MAPK) pathway, which is induced by pro‐inflammatory stimuli [114]. This pathway mediates cytoskeletal re‐arrangements in endothelial cells upon exposure to ROS [115], and is also involved in leukocyte TEM [116]. One of the substrates of the p38 MAPK pathway that mediates these effects is Leukocyte‐Specific Protein‐1 (LSP1) [117]. Originally identified in leukocytes, this protein binds intracellular calcium and regulates the cortical actin rim with an F‐actin‐binding domain [118]. In endothelial cells, LSP1 plays a crucial role in leukocyte TEM [119], although this effect has not been proven in all inflammation models [120], most likely reflecting physiological differences between the microvasculature of different organs. Nevertheless, it was established that LSP1 induces cytoskeletal rearrangements to initiate endothelial dome formation and prevent vascular leakage during leukocyte TEM [121]. Similar to its upstream activator MAPK‐activated protein kinase 2 (MK2), LSP1 resides in the nucleus under resting conditions but is transported to the cytoskeleton upon inflammation [122]. This provides a strategy to prevent cytoskeletal rearrangements until they are critically needed in the endothelial cell (Figure 3a).

### 8.2 Ventral Lamellipodia

Ventral lamellipodia provide another mechanism that prevents vascular leakage specifically after the completion of leukocyte TEM. The first cues for this mechanism were found during live imaging of an in vitro inflammation model, revealing that paracellular and transcellular gaps were closed within minutes after leukocyte TEM by an F‐actin‐rich structure that pushed the membrane forward in a lamellar fashion [123]. The origin of these lamellar protrusions was found on the basolateral (ventral) surface of the endothelial cell, where after these F‐actin‐rich structures were referred to as ventral lamellipodia (VL). Considering their morphology, VL show similarities to F‐actin‐rich ventral waves (VW) [124], which are elusive structures involved in neutrophil migration [125]. Although VL and VW both form F‐actin‐driven membrane protrusions, VW typically form membrane protrusions that bridge the extracellular space and stop propagating when they reach a cell membrane [126], whereas VL often continue their propagation after the bridging step is completed [123].

The mechanism by which VL prevent vascular leakage during leukocyte TEM was shown to be dependent on stress fibers. Following leukocyte TEM, a regional subset of stress fibers undergoes physical retraction, which is indicative of a local decrease in isometric tension of the cytoskeleton [127]. Due to this retracted state, such stress fibers become so‐called parent actin filaments that accommodate VL nodes for the initiation of VL [123]. Interestingly, this response is solely driven by the mechanical force of leukocyte TEM, as artificial micro‐wounds are sufficient to elicit VL formation as well. After their initiation, VL propagate toward transcellular or paracellular pores to bridge them, typically in an asymmetric fashion from one side of the pore to the other. Intriguingly, VL are initiated in adjacent and non‐adjacent endothelial cells as well, presumably to bridge potential gaps at the endothelial junctions that can also occur due to the mechanical force of leukocyte TEM. Another fascinating feature of VL is that when they are initially oriented in the wrong direction, they are capable of steering in the direction of the pore. Altogether, this shows that VL have intricate abilities to sense both local and distant cytoskeletal discontinuities in endothelial cells, which are caused by the mechanical force of a leukocyte during TEM. However, a permeability assay to test the direct effect of VL on vascular leakage during TEM has not been conducted.

Rac1 plays a pivotal role in the formation of VL. Previously identified as one of the small Rho GTPases involved in actin cytoskeletal rearrangements, Rac1 facilitates the formation of membrane ruffles and lamellipodia in endothelial cells [47]. In migrating leukocytes, the leading front prevents actin polymerization in other cellular regions by increasing their cellular tension, which inhibits actin polymerization by Rac1 [128]. This observation led to the hypothesis that Rac1‐driven actin polymerization is activated in regions of reduced cellular tension, coinciding with the sites where VL emerge. Consistent with this hypothesis, Rac1 enrichment has indeed been observed at the initiation node and leading edge of VL [123]. Moreover, Rac1 enrichment colocalizes with p47phox, a subunit of the NAD(P)H oxidase [129] that is activated by Rac1 and produces ROS [130]. These ROS are produced in close proximity to the actin cytoskeleton, as IQGAP and cortactin are also enriched at VL initiation nodes and leading edges [123]. Both these molecules link the NAD(P)H oxidase to the cytoskeleton [131, 132].

In this way, local ROS production may result in the recruitment of Arp2/3 to the initiation node and leading edge of VL, triggering actin‐driven propagation of VL [133] (Figure 3b). This process is similar to the ROS‐dependent actin polymerization during dorsal ruffle formation [134].

### 8.3 F‐Actin‐Rich Contractile Ring

Upon the initial start of the diapedesis, known as the early diapedesis step, the endothelium initiates the formation of an actin belt around the adhering leukocyte. This actin belt, characterized by a ring of F‐actin, is observed around every leukocyte type tested and involves RhoA signaling. It is believed that this ring provides another mechanism that prevents vascular leakage during leukocyte TEM [135]. However, from a traditional point of view, RhoA is known to induce focal adhesions that serve as anchoring points between stress fibers of the cytoskeleton and the extracellular matrix [40]. The observation that E‐selectin and ICAM‐1 also associate with the cytoskeleton [136, 137] opened the door for the involvement of RhoA as the potential linker between these adhesion receptors and the endothelial cytoskeleton. Indeed, it was proven that when leukocytes bind to the endothelium, RhoA promotes firm adhesion by clustering E‐selectin, ICAM‐1 and VCAM‐1 on the membrane via the cytoskeleton [138]. Vice versa, crosslinking ICAM‐1 also activates RhoA in a positive feedback loop [139, 140]. Based on the finding that RhoA also induces contraction of stress fibers [43], it was assumed that leukocyte docking on endothelial cells during TEM automatically opens the lateral junction for diapedesis via RhoA‐mediated contraction of stress fibers.

The mechanism by which RhoA functions and prevents vascular leakage during leukocyte TEM, however, was found to be quite different from this original role. In fact, a later study established that RhoA is not involved in the adhesion of leukocytes, but in the diapedesis step of leukocyte TEM [135]. Biosensor experiments using FRET‐based RhoA probes allowed for the spatial and temporal resolution of RhoA activation and showed that endothelial RhoA was activated during the diapedesis step, but not during the adhesion step. Moreover, RhoA was found to act rather locally than globally during this process in the endothelial cell, because it colocalized with the F‐actin‐rich ring around the transmigrating leukocyte. These actin rings highly colocalized with phospho‐MLC, as was determined by using antibodies that specifically recognized this active state of MLC, indicating that the F‐actin ring was under actomyosin‐driven tension. In addition, RhoA activates ROCK2b, a Rho‐associated protein kinase [42] that phosphorylates MLC on the F‐actin‐rich ring in an asymmetric fashion. Like an elastic strap, this phosphorylation exerts a contractile force on the F‐actin ring, confining the pore diameter of the ring around the migrating leukocyte during diapedesis and closing the pore after diapedesis is completed, thereby preventing vascular leakage. Other contributors to the contraction of this tensile F‐actin‐rich ring are the RhoGEFs leukemia‐associated Rho guanine nucleotide exchange factor (LARG) and Ect2. These RhoGEFs are likely to be recruited to the intracellular tail of ICAM‐1 by the mechanical force of leukocyte docking [141]. Subsequently, they contribute to the contraction of the F‐actin‐rich ring, which is not surprising as Ect2 is also found at contractile rings during cytokinesis [142] (Figure 3c).

ICAM‐1 is thus not only involved in the adhesion of leukocytes via integrins, but also in the prevention of vascular leakage during the diapedesis step of leukocyte TEM, as it recruits the RhoGEFs LARG and Ect2 to induce the tensile F‐actin‐rich ring. Importantly, ICAM‐1 is also present in F‐actin‐rich apical protrusions, observed in 40% of the diapedesis events [135]. Formation of these protrusions is not dependent on RhoA, but instead requires RhoG which is activated by ICAM‐1 clustering on the endothelial membrane [143]. Interestingly, so‐called endothelial TEM hotspots that prevent vascular leakage during leukocyte TEM [144] are also enriched in ICAM‐1 [144]. In fact, the first three extracellular domains of ICAM‐1 have been denoted as recognition markers for leukocytes at endothelial TEM hotspots. This is believed to safeguard the endothelial barrier by restricting leukocyte TEM to specific endothelial regions, thus preventing widespread disruptions of the endothelium. Moreover, endothelial hotspots might be linked to downstream machinery that prevents vascular leakage during leukocyte TEM, like the F‐actin‐rich ring that is also induced by ICAM‐1 [135]. However, de‐activation of downstream ICAM‐1 signaling did not reduce the barrier‐protective effects of endothelial hotspots, which implies that they operate uncoupled from downstream F‐actin‐rich rings.

### 8.4 GPVI Receptor

Platelets were traditionally considered the primary mediators of coagulation following vascular injury, but their involvement in multiple other processes has currently been well established [145]. Glycoprotein VI (GPVI) is a collagen receptor exclusively expressed on platelets that has been shown to prevent vascular leakage during leukocyte TEM. Located on the membrane, GPVI forms a complex with the Fc receptor γ‐chain that contains an immunoreceptor tyrosine‐based activation motif (ITAM) [146]. After binding its ligand collagen, GPVI phosphorylates this motif to activate the downstream kinases spleen tyrosine kinase (Syk) [147] and Bruton's tyrosine kinase (Btk) [148]. Together, these kinases mediate the degranulation of serotonin and aggregation of platelets via phospholipase C γ2 (PLCy2).

The mechanism that explains how GPVI on platelets prevents vascular leakage in inflamed vessels was first hypothesized to be dependent on the attenuation of leukocyte cytotoxicity [149]. Surprisingly, it was found that GPVI increases leukocyte degranulation of matrix metalloproteinase‐9 (MMP‐9) and the production of ROS, which are known mediators of leukocyte cytotoxicity. However, such a stimulatory effect of platelets on leukocyte cytotoxicity has been described in earlier research [150]. As an alternative hypothesis, it was suggested that platelets use GPVI to bind the collagenous basement membrane at regions where endothelial cells are disrupted and the vascular barrier is compromised. Using intravital microscopy, it was established that platelets indeed adhere to exposed parts of the basement membrane in inflamed vessels [149]. Interestingly, leukocytes that possess ligands for GPVI can be used to be recruited to platelets at inflamed vessels [151].

### 8.5 Tie‐2

Tie‐2 is a receptor tyrosine kinase on endothelial cells, and is involved in the remodeling of newly formed blood vessels during embryonic development [152]. The activity of Tie‐2 is regulated by VE‐PTP, which associates with Tie‐2 on the endothelial membrane and continuously dephosphorylates it, thereby inhibiting its activity [153]. Genetic deletions of VE‐PTP domains lead to lethal vascular remodeling defects in embryonic mice [154], caused by excessive Tie‐2 activation, which triggers endothelial proliferation and vessel enlargement [155]. Therefore, strict regulation of Tie‐2 is crucial for maintaining its beneficial effects. One of the beneficial effects of Tie‐2 is its ability to prevent vascular leakage during inflammation upon binding of its ligand, angiopoietin‐1 (Ang1) [156]. Moreover, inhibition of VE‐PTP also prevents vascular leakage during inflammation [157], which is mediated by an increase in Tie‐2 activity [158]. This highlights a paradoxical role of VE‐PTP, as it protects the endothelial barrier through the dephosphorylation of VE‐cadherin [72] but also undermines the endothelial barrier by suppressing Tie‐2 activity. Importantly, the activation of Tie‐2 after VE‐PTP inhibition resulted in a stronger endothelial barrier than the dephosphorylation of VE‐cadherin by VE‐PTP under normal circumstances [158].

The mechanism that explains how Tie‐2 prevents vascular leakage during leukocyte TEM was shown to be dependent on the binding of its ligand Ang1 [159]. More precisely, platelets that adhere to Von Willebrand Factor (VWF) on the inflamed endothelium were found to be an important source of Ang1 for endothelial cells [160]. Upon the binding of Ang1, the Tie‐2 receptor activates several downstream effectors, including the GTPases Rap1 and Rac1. Both these GTPases induce cytoskeletal rearrangements to protect the endothelial junction and prevent stress fiber formation [161, 162]. To be specific, Rap1 exerts this effect via the recruitment of the guanine nucleotide exchange factor (GEF) FGD5 to the endothelial junction, which is known as a Cdc42‐GEF [163]. At the endothelial junction, FGD5 becomes phosphorylated by Tie‐2 and contributes to cytoskeletal rearrangements that result in the formation of an F‐actin‐rich ring around the migrating leukocyte [135, 160]. In this way, Ang1 produced by platelets eventually contributes to the prevention of vascular leakage during leukocyte TEM (Figure 3e).

### 8.6 Cooperation of Different Mechanisms

Although the previous five mechanisms are presented as separate entities, it is plausible that they cooperate as a unified system to prevent vascular leakage during leukocyte TEM. Moreover, a functional overlap may exist between the different mechanisms. For instance, a follow‐up study showed that the endothelial dome regulator LSP1 [121] is activated downstream of ICAM‐1 clustering [164]. Since the RhoA‐dependent F‐actin‐rich contractile ring is also dependent on ICAM‐1 clustering [135], it is not unthinkable that LSP1 and RhoA function as a coordinated system, using ICAM‐1 clustering as a shared upstream trigger to control vascular leakage. In this scenario, ICAM‐1 clustering may activate LSP1 to form an endothelial dome first, after which LSP1 activates RhoA to induce an F‐actin‐rich contractile ring. However, further research is needed to establish a direct causal relationship between LSP1 and RhoA. Another striking similarity between the proposed mechanisms is the asymmetric closure of pores, which occurs in the F‐actin‐rich contractile ring [135], but also in pore closure by VL [123]. Although the F‐actin‐rich contractile ring is formed during diapedesis and VL is typically activated after diapedesis is completed, it is remarkable that both systems use an asymmetric pore closure system, while one might expect a purse string mechanism [165]. Future studies are needed to delineate whether asymmetric dynamics of closing systems result in more efficient pore closure. Finally, it seems plausible that platelets adhering to the basement membrane via the GPVI receptor also produce Ang1 [149], similar to platelets that adhere to VWF on the inflamed endothelium [160]. Although this has already been confirmed in a later study [166], future research is needed to elucidate if GPVI‐mediated Ang1 production also regulates the F‐actin‐rich ring.

## 9 Clinical Implications

In this section, we will highlight the various in vitro and in vivo models that have been developed to mimic human leukocyte TEM and vascular leakage in the laboratory. Subsequently, we highlight medical treatments that have been derived from these research models.

## 10 Laboratory Models to Mimic Leukocyte TEM

### 10.1 In Vitro Models

The first in vitro models that were created to simulate leukocyte TEM were static, meaning they did not account for the shear stress exerted by blood flow through a vessel. In these models, endothelial cells are grown on a scaffold that separates an upper compartment from a lower compartment, representing the apical side and the basal side of the endothelium, respectively [167]. To prime the endothelium for interactions with leukocytes, endothelial cells are usually treated with pro‐inflammatory mediators, although interactions can also occur in the absence of this priming step, which is often omitted in control experiments [168]. Subsequently, leukocytes are introduced to the apical upper compartment, while chemoattractants are added to the basal lower compartment to create a chemotactic gradient. Following this step, leukocyte TEM can be quantified by counting the leukocytes in the basal compartment. Moreover, vascular leakage can be assessed by various methods, including dextrans labeled with fluorescent isothiocyanate (e.g., FITC‐Dextran) as a key method [169].

Building on the static assay, the flow chamber TEM assay emerged as a second in vitro model to study leukocyte TEM. This method introduced flow conditions on the apical side of the endothelium to capture the dynamic interactions between leukocytes and the endothelial wall [170]. While this model resembles the in vivo circumstances more accurately than static transmigration assays, its scaffolds are still relatively stiff and lack surrounding tissue that is present in vivo [167]. A potential solution to these limitations is the organ‐on‐a‐chip model, a microfluidic cell culture system that offers a highly realistic, three‐dimensional representation of the in vivo physiology [171]. This model allows researchers to control environmental factors such as shear stress and pressure, to perform high‐resolution real‐time imaging, and to integrate organs on different chips. Moreover, it enables the use of patient‐derived cells, offering promising applications in personalized medicine [172]. Since the establishment of the first blood‐vessel‐on‐a‐chip model [173], this system has been used to study leukocyte TEM and holds great potential for advancing our understanding of this process [174].

### 10.2 In Vivo Models

One of the first in vivo models to study leukocyte TEM is the peritonitis model, in which mice receive intraperitoneal injections of thioglycollate to induce a non‐infectious peritonitis [175]. In this model, leukocyte TEM is typically assessed by counting the leukocytes present in the peritoneal exudate. Another organ that is frequently used to assess leukocyte TEM is the skin. Dermal inflammation can be induced by direct injection of chemicals [176], induction of immune complexes using the reverse passive Arthus reaction [177] or induction of T‐cell infiltration using the delayed‐type hypersensitivity method [178]. In this model, leukocyte TEM can be appreciated by taking skin biopsies to count transmigrated leukocytes. A third in vivo model to study leukocyte TEM is the cremaster muscle, in which inflammation can be induced by intrascrotal injection of pro‐inflammatory agents [179] or temporal clamping of arteries [180], the latter of which induces ischemia–reperfusion inflammation. The cremaster muscle offers a highly accessible and transparent model, making it particularly suitable for intravital imaging [181]. Using this model, leukocyte TEM can be appreciated by real‐time counting of leukocytes outside the vascular barrier. For all three described in vivo models, vascular leakage can be assessed using various methods, including FITC‐dextran [169].

## 11 Potential Targets in Human Medicine

### 11.1 Sepsis

Sepsis is a life‐threatening disease that affects more than 50 million people each year, and results in 5.3 million annual deaths worldwide [182]. By definition, sepsis is a dysregulated response of the human body to an infection, which leads to a life‐threatening condition of organ dysfunction [183]. One of the hallmarks of sepsis is endothelial barrier dysfunction, which results in tissue oedema and decreased perfusion of organs [184]. Although the importance of vascular leakage in sepsis is well established, no therapies directed at vascular leakage are implemented in clinical practice to date [185]. Among potential therapies, activated protein C (APC) has been thoroughly investigated. This protein circulates in the blood and binds endothelial cells, whereafter it is activated by thrombin to suppress local coagulation [186]. Moreover, APC also reinforces the endothelial barrier via cytoskeletal rearrangements mediated by Rho GTPases. Due to its anticoagulant and barrier‐protective features, APC was temporarily accepted by the European Medicines Agency for the treatment of sepsis [187]. However, APC was later withdrawn from the market due to disappointing results in terms of mortality [188]. Another therapeutic that holds potential for treating sepsis is the Abl‐related gene kinase (Arg/Abl2) imatinib, which prevents vascular leakage by reinforcing cell‐matrix adhesions via Rac1 [189]. Clinical trials are needed to fully assess the potential benefits of imatinib in treating sepsis. Furthermore, Ang1 has been introduced as a prognostic biomarker that predicts lower mortality in sepsis patients [190]. Given its endothelial barrier‐protective effects in vivo [160], it is worth investigating if Ang1 can be used beyond the scope of diagnostics as a therapeutic for sepsis.

### 11.2 COVID‐19

The COVID‐19 pandemic led to an estimated 18.2 million deaths worldwide between January 2020 and December 2021 [191]. COVID‐19 typically manifests as pneumonitis, which can progress to acute respiratory distress syndrome (ARDS). One of the defining hallmarks of COVID‐19 is endothelial injury, which is caused by the virus itself, and leads to diffuse pulmonary edema [192, 193]. Because the pathophysiology of endothelial damage in COVID‐19 shows similarities with sepsis, the Arg/Abl2 inhibitor imatinib has also been enrolled in a clinical trial for COVID‐19 [194]. Although this study failed to prove a significant improvement in mortality, the median duration of mechanical ventilation was significantly shorter in patients treated with imatinib, suggesting that imatinib may effectively reduce vascular leakage in COVID‐19. Another promising therapeutic candidate is Bß15‐42, also known as FX06 [195]. In biology, FX06 is traditionally known as a degradation product of fibrin degradation. However, FX06 also stabilizes VE‐cadherin on endothelial junctions and prevents endothelial cell contraction, thereby reducing vascular leakage. Against expectations, a clinical trial failed to show a reduction of vascular leakage in COVID‐19 following treatment with FX06, which was measured by the extravascular lung water index [196]. However, it must be noted that this study had a low sample size and only included patients that were already on mechanical ventilation with ARDS. Therefore, a study with earlier administration and a larger sample size is necessary to fully assess the potential of FX06 for the treatment of COVID‐19.

### 11.3 Atherosclerosis

Atherosclerosis is a chronic inflammatory disease responsible for 75% of all cardiovascular diseases, which collectively lead to 18.6 million worldwide deaths each year [197]. Cardiovascular risk factors are the primary cause of atherosclerosis, the traditional ones being hypercholesterolemia, hypertension, and smoking [198]. Atherosclerosis starts with the activation of endothelial cells by lipid mediators, which triggers leukocyte TEM and initiates the formation of an atherosclerotic plaque [199]. As the plaque expands, chronic production of pro‐inflammatory agents results in the formation of new blood vessels that grow into the plaque [13]. These vessels typically have a limited barrier function and lead to intraplaque hemorrhage, which increases the risk of plaque rupture and thrombus formation in the blood vessel. Although endothelial dysfunction clearly plays a key role in atherosclerosis, there are currently no therapies available that specifically target this issue. The primary treatment for atherosclerosis involves statins, which reduce LDL cholesterol levels in the plasma [200].

Interestingly, statins have also been shown to reduce leukocyte TEM by inhibition of LFA‐1, which may account for some of their therapeutic benefits [201]. In line with this, it would be valuable to explore in clinical trials whether targeting additional mediators of leukocyte TEM, such as selectins [202], ICAM‐1 [203], VCAM‐1 [204], and PECAM‐1 [205] could provide further therapeutic benefits in atherosclerosis. Another promising strategy is the use of colchicine, which has been shown to lower the incidence of cardiovascular events, possibly through its effects on leukocyte TEM [206]. In addition to strategies that reduce leukocyte TEM in atherosclerosis, aimed at preventing vascular leakage by decreasing the chronic inflammatory conditions, targeting the fragile new blood vessels within atherosclerotic plaques could also offer potential therapeutic benefits. In this light, the Rho kinase inhibitor fasudil might have therapeutic potential, as it enhances endothelial function in patients with coronary artery disease [207]. In addition, the transcription factor hypoxia‐inducible factor 1a (HIF‐1a) might be an interesting therapeutic target. HIF‐1A is activated in macrophages residing in the atherosclerotic plaque, and stimulates the production of VEGF to form new blood vessels that have a fragile character [208]. Moreover, VEGF also triggers the endocytosis of VE‐cadherin in endothelial cells, resulting in additional vascular leakage [209]. Further in vivo studies are needed to explore whether fasudil and HIF‐1a inhibitors are promising candidates to limit vascular leakage within atherosclerotic plaques.

### 11.4 Inflammatory Bowel Disease

IBD is an increasingly common global disease, currently affecting 0.3% of the human population [210]. Classified as an autoimmune disorder, IBD is marked by episodes of severe inflammation in the gastrointestinal tract, which can be potentially life‐threatening [211]. In clinical practice, IBD typically presents as either Crohn's disease or ulcerative colitis [212]. Notably, Crohn's disease may impact any part of the gastrointestinal tract, whereas ulcerative colitis is generally limited to the distal colon. Although the exact pathophysiology of IBD remains to be elucidated, it is believed that immune responses to microbes in the gut play a central role [213]. These immune responses are illustrated by massive leukocyte TEM toward the intestinal tissue, inducing a chronic inflammatory condition that waxes and wanes [214]. Similar to atherosclerosis, chronic local production of pro‐inflammatory agents induces angiogenesis via growth factors such as VEGF, giving rise to fragile blood vessels that result in vascular leakage [14]. While broad‐acting immunosuppressants like corticosteroids were once the pillar of IBD treatment [215], recent innovations have introduced biologicals designed to target specific components of the immune response [216]. These biologicals include infliximab targeting TNFα [217, 218], ustekinumab targeting interleukins [219, 220] and vedolizumab targeting integrins [221, 222]. Interestingly, vedolizumab specifically targets the α4ß7 integrin on lymphocytes [223], which binds to mucosal addressin‐cell adhesion molecule 1 (MAdCAM‐1) on endothelial cells [224]. As MAdCAM‐1 is exclusively expressed on endothelial cells of the intestine, it represents a highly selective target for modulating leukocyte TEM within the gut. Similar to atherosclerosis, HIF‐1a is also involved in the angiogenesis of leaky blood vessels in IBD [225], making it an interesting therapeutic target for IBD that warrants further investigation in future studies.

## 12 Conclusion

Transendothelial migration (TEM) of leukocytes is a key step during inflammation, where leukocytes traverse the endothelial barrier to reach sites of tissue damage. In this review, we have highlighted five mechanisms that prevent vascular leakage during TEM. These mechanisms likely operate in concert as a cohesive system to limit vascular leakage under inflammatory conditions that may become derailed upon chronic inflammatory conditions. Future research should focus on integrating these mechanisms into a comprehensive framework, which could provide deeper insights into the regulation of vascular leakage. This understanding has the potential to inform therapeutic strategies for diseases characterized by excessive vascular leakage, including acute inflammatory conditions such as sepsis and COVID‐19, as well as chronic inflammatory disorders like atherosclerosis and IBD.

## Conflicts of Interest

The authors declare no conflicts of interest.

## Data Availability

The data that support the findings of this study are available on request from the corresponding author. The data are not publicly available due to privacy or ethical restrictions.
